# Inhibitory Effect of Black and Red Pepper and Thyme Extracts and Essential Oils on *Enterohemorrhagic Escherichia coli *and DNase Activity of *Staphylococcus aureus*

**Published:** 2013

**Authors:** Maryam Zarringhalam, Jalal Zaringhalam, Mehdi Shadnoush, Firouzeh Safaeyan, Elaheh Tekieh

**Affiliations:** a *Department of Microbiology, International Branch of Shahid Beheshti Medical University, Tehran, Iran.*; b *Department of Physiology, Neuroscience Research Center, Shahid Beheshti University of Medical Sciences, Tehran, Iran.*; c *Food Science and Technology Department, Nutritional Institute, Shahid Beheshti University of Medical Sciences, Tehran, Iran.*; d *Department of Microbiology, Tabriz University of Medical Sciences, Tabriz, Iran*

**Keywords:** *Escherichia coli *O157: H7, *Staphylococcus aureus*, DNase activity, Black pepper, Red pepper, Thyme

## Abstract

In this study, extracts and essential oils of Black and Red pepper and Thyme were tested for antibacterial activity against *Escherichia coli O157: H7 *and *Staphylococcus aureus*. Black and Red pepper and Thyme were provided from Iranian agricultural researches center. 2 g of each plant powder was added to 10 cc ethanol 96°. After 24 h, the crude extract was separated as an alcoholic extract and concentrated by distillation method. Plants were examined for determining their major component and essential oils were separated. Phytochemical analyses were done for detection of some effective substances in extracts. The antibacterial activity against *Escherichia coli O157: H7 *and *Staphylococcus aureus *was tested and the results showed that all extracts and essential oils were effective and essential oils were more active. The extracts and oils that showed antimicrobial activity were later tested to determine the Minimum Inhibitory Dilution (MID) for those bacteria. They were also effective on the inhibition of DNase activity. This study was indicated that extracts and essential oils of Black and Red pepper and Thyme can play a significant role in inhibition of *Escherichia coli O157:* *H7 *and *Staphylococcus aureus.*

## Introduction

*Staphylococcus aureus *is one of the most frequently identified pathogens in clinical laboratories and DNase is it’s an important virulence factor. DNase expression allows *Staphylococcus aureus *to escape killing in neutophils extracellular traps. Infections caused by *S. aureus *range from minor skin disorders such as wound infections, furuncles and carbuncles, and bullous impetigo, through locally invasive diseases such as cellulitis, osteomyelitis, sinusitis, and pneumonia, to major life-threatening septicemia and meningitis (1). This bacterium is also a frequent cause of medical device-related infections such as intravascular line sepsis and prosthetic joint infections. Although minor skin infections may resolve naturally without antibiotic intervention, once *S. aureus *invades deeper structures, it often spreads hematogenously to other organ systems, leading to metastatic infection. Endocarditis and septicemia have significant morbidity and mortality despite aggressive antimicrobial therapy. *Staphylococcal *food poisoning occurs with a short incubation period of 2-6 h and is characterized by nausea and vomiting, that is followed by abdominal cramps and diarrhea, which can be hemorrhagic. It is mediated by enterotoxin B and occurs due to ingestion of food contaminated with preformed toxins (2).

*Escherichia coli *is a member of the normal flora of the human and animal gastrointestinal tract, and its several pathogenic types can cause different diseases. *E. coli O157:H7 *and other Shiga toxin-producing *E. coli *(STEC) strains have emerged in recent years as important human pathogens associated with a spectrum of diseases ranging from diarrhea to hemorrhagic colitis and hemolytic-uremic syndrome (HUS). Due to the morbidities and mortalities associated with outbreaks and sporadic cases of STEC diseases, these pathogens are now considered as major public health problems of worldwide importance. *E. coli O157:H7 *is a serotype most frequently isolated from patients, and shares a variety of virulence factors, including two Shiga toxins, Stx1 and Stx2, and a pathogenicity island, termed as the locus for enterocyte effacement that encodes the proteins responsible for the intimate adherence of *E.* *coli *to epithelial cells. The production of Shiga toxins by *E .coli O157:H7 *has a major role in pathogenesis, particularly in the pathogenesis of HUS*.*

There are several antibacterial agents that can prevent the production of this enzyme (1, 3). *Staphylococcus aureus *and *E. coli O157:H7* can develop drug resistance to many chemical

drugs. Thus, considerable effort has been expanded by investigators in the development of herbal drugs. Scientific experiments since the late 19^th^ century have documented the antimicrobial properties of some spices, herbs, and their components*. *Studies confirm that the growth of both gram-positive and gramnegative food borne bacteria can be inhibited by some herbs (1, 4). Extracts of Black and Red pepper and Thyme are traditionally used for the treatment of infectious diseases. 

*Thymus vulgaris *(Common thyme) is a member of lamiaceae family, which distributes in areas of Mediterranean, Asia and is cultivated in all over the world including Iran. Common thyme contains 0.8-2.6 % volatile oil consisting of highly variable amount of phenols, monoterpene hydrocarbons, and alcohols. Thymol is normally the major phenolic component in common thyme. Thyme is used as an antispasmodic, carminative, antiseptic, antimicrobial and natural food preservative (5, 7). Pepper is a member of Piperaceae family and contains some of the antimicrobial components such as Terpinene, *α*-pinene, *β*-pinene, Linaleol and Terpineol (8, 9). Capsaicin is the major active chemical compound of peppers (8-methyl-*N*-vanillyl-6-nonenamide). Capsaicin and several related compounds are called capsaicinoids and are produced as secondary metabolites by chili peppers, probably as deterrents against herbivores. Pure capsaicin is a hydrophobic, colorless, odorless, and crystalline to waxy compound (10). Capsaicinoids have various physiological and pharmacological effects on the gastrointestinal tract motility, cardiovascular and respiratory system as well as the sensory and thermoregulation system. These effects result principally from the specific action of capsaicinoids on primary afferent neurons of the C-fiber type (11). The aim of this study was to investigate the inhibitory effect of Black and Red pepper and Thyme extracts and their essential oils on the *Enterohemorrhagic Escherichia coli *and DNase activity of *Staphylococcus aureus.*

## Experimental

*Preparation of materials*

Black and Red pepper and Thyme were provided by the Iranian agricultural researches center. After drying, 2 g of the plant powder was added to 10 cc ethanol 96˚. After 24 h, the crude extract was separated as an alcoholic extract and concentrated according distillation method. The dried or fresh herbs were combined with alcohol, the solid matter was then removed leaving only the oils of the herbs mixed with the alcohol. When fresh herbs were used, the most common ratio is 1:1. Dry herb strength 1:5 meant that the mixture used to produce the extract was 1 part dried plant and 5 parts ethanol 96° (11).

**Table 1:** Inhibitory effect of extracts and oils on growth of *Staphylococcus aureus*

**Plant extracts**	**Diameter zone of inhibition (mm)**
Alcoholic extract of Thyme	14
Alcoholic extract of Black pepper	14
Alcoholic extract of Red pepper	15
Alcohol	-
Thyme oil	28 ■,□, ▲▲▲
Black pepper oil	24 ○○○
Red pepper oil	25 ●●●

Diameters of the inhibition zone are given in mm. Alcohol was inactive. ■ p < 0.05: for comparing Thyme oil with Black pepper oil.** □ **p < 0.05: for comparing Thyme oil with Red pepper oil. ▲▲▲ p < 0.001: for comparing Thyme oil with Alcoholic extract of Thyme.

*Phytochemical analysis Thyme*

Thyme plant was analyzed for determining and quantifying phenolic compounds including thymol and carvacrol. British Pharmacopiea method under thyme monograph was used. Using Gas Chromatography with capillary column, quantization of phenolic compounds was done (12).

*Black pepper*

Gas chromatography/ mass spectrometry was used to determine components using a 0.3 m × 0.25 mm ID (0.20 μm film thickness) (Agilent 7890N) and Supelco SP-2330 capillary column (Supelco, Inc., Bellefonte, PA, USA). One micro liter was injected by an auto sampler into the chromatograph, equipped with a split injector and a Flame Ionization Detector (FID). The split ratio was 1:20 after injection of 1 μL of the Fatty Acid Methyl Esters (FAME). The injector temperature was programmed at 250°C and the detector temperature was programmed at 300°C. The column temperature program initiated ran at 100°C, for 2 min, warmed up to 170°C at 10°C /min, hold for 2 min, warmed up to 200°C at 7.5°C /min, and then holds for 20 min to facilitate optimal separation. The GC mass analysis was carried out to determine the major components of the extractions on a Shimadzu GCMS-QP2010 Plus equipped with a BPX-5 column (30 m × 0.25 mm × 0.25 μm), helium as carrier gas at a flow rate of 1 mL min-1, in electronic impact mode (70 eV) and split injection ratio (1:20). The injector and GC/MS interface were kept at 320°C. The column temperature program was as follows:

50°C, heating at 10°C min-1 until 320°C and remaining at this temperature for 15 min. The components of the oils were identified by comparison of the mass spectra with the NIST08 library information (11).

*Red pepper*

Total phenolic content (TPC) was estimated as gallic acid equivalents (GAE) as described by Folin–Ciocalteau’s (FC) method with modifications (13). An aliquot (0.5 mL) of the pepper extract solution was transferred to a glass tube; 0.5 mL of reactive FC was added after 5 min; 2 mL of Na_2_CO_3_ (200 g/L) were added and shaken. After 15 min of incubation at ambient temperature, 10 mL of ultra-pure water was added and the formed precipitate was removed by centrifugation during 5 min at 4000×g. Finally, the absorbance was measured in a spectrophotometer (Spectronic_20 GenesysTM, Illinois, USA) at 725 nm and compared to a GA calibration curve. Results were expressed as mg acid gallic/100 g dry matter. All reagents were purchased from Merck (Merck KGaA, Darmstadt, Germany), and all measurements were done in triplicate (13, 14).

*Strain of bacteria*

*Escherichia coli O157: H7 (EHEC) *and strain of *Staphylococcus aureus *(ATCC 29213) was obtained from the Reference laboratory of Iran (Tehran, Iran). This work has been performed in the Department of Food Science and Technology, in Sofyan Azad University (Iran) in 2010.

*Determination of inhibitory effect of extracts on Escherichia coli O157: H7 and Staphylococcus aureus*

The inhibitory effect of extracts and essential oils were tested by the agar - well – diffusion assay. 5 mm - diameter wells were made on agar media which were preinoculated with *Escherichia coli O157: H7 *and *Staphylococcus* *aureus *and each well was filled with 50 μL of each extracts and oils. Inhibition zones around the wells were measured and recorded (11).

*Determination of minimum inhibitorydilutions*

The extracts and oils that showed antimicrobial activity were later tested to determine the Minimum Inhibitory Dilution (MID) for *Escherichia coli O157: H7 *and *Staphylococcus aureus*. Bacterial sample was grown in Muller Hinton broth for 6 h. After that, 1 mL of 10^6^ cells was inoculated in tubes containing Muller Hinton broth, supplemented with different dilutions (1:2-1:64) of the extracts oils. After incubation for approximately 18 h at 37°C, the lowest dilution in the tube showing visual inhibition of growth was the minimum inhibitory dilution (11).

*Evaluation of extracts DNase activity inhibitory effects*

Sub- Minimum Inhibitory Dilutions of extracts and oils were used to evaluate the DNase activity in *Staphylococcus aureus*. 10 μL of these suspensions was plated separately on DNase agar. After 24 h incubation, zone of DNase was assayed by adding of 1N HCl (11).

*Statistical analysis*

The data were analyzed using General Linear Model (GLM). Student‘s t-test was used to compare means. Data are presented as mean± SEM. Significance level for the comparison of the group means was set at p < 0.05.

*Discussion and results*

In the present study, we first examined plants for determining major components. Thyme showed 2.2 % (w/w) of essential oil which was rich in phenolics (as thymol and carvacrol) comprising 63.0% of total oil. Results revealed that the major components of *P. nigrum* extract contained piperine (74.34%), oleic acid (40.67%), linoleic acid (34.17%), caryophyllene (18.53%) and palmitic acid (18.03%). Total phenolic content of Red pepper was 370 mg galic acid/100 g dry matter. In the next step of this study, the alcoholic extracts and oils of Black and Red pepper and Thyme were tested for antibacterial activity against *Escherichia coli* *O157: H7 *and *Staphylococcus aureus *(Table1,2). 

**Table 2:** Inhibitory effect of extracts and oils on growth of *Escherichia coli O157:H7*

**Plant extracts**	**Diameter zone of inhibition (mm)**
Alcoholic extract of Thyme	15
Alcoholic extract of Black pepper	13
Alcoholic extract of Red pepper	13
Alcohol	-
Thyme oil	29 ○○,●●,▲▲▲
Black pepper oil	23 □□□
Red pepper oil	23 ■■■

Diameters of the inhibition zone are given in mm. Alcohol was inactive.

○○ p < 0.01: for comparing Thyme oil with Black pepper oil.

●● p < 0.01: for comparing Thyme oil with Red pepper oil.

▲▲▲ p < 0.001: for comparing Thyme oil with Alcoholic extract of Thyme.

□□□ p < 0.001: for comparing Black pepper oil with Alcoholic extract of Black pepper. ■■■p < 0.001: for comparing Red pepper oil

with Alcoholic extract of Red pepper.

All the tested plants were significantly active against *Escherichia coli O157: H7 *and *Staphylococcus aureus*. Moreover, the results indicated that, antimicrobial activity of Thyme is stronger than Black pepper and red pepper, which showed the highest inhibition zone (p < 0.05 for *Staphylococcus aureus *and p < 0.01 for *Escherichia coli O157:H7)*. We found no significant difference between Black Pepper and Red pepper anti-*Staphylococcus *and anti- *Escherichia coli O157:H7 *activity. Our results also stated that oils of those plants indicated more antimicrobial effects than their alcoholic extracts. Then, as reported by previous, some studies it seems that the oils antimicrobial components of herbs are different than alcoholic extracts (15). 

Furthermore, the extracts and oils which showed antimicrobial activity were tested to determine minimum inhibitory dilution (Table 3, 4). 

**Table 3:** Determination of minimum inhibitory dilution of extracts for *Staphylococcus aureus *(ATCC 29213).

**Plant extracts**	**Minimum Inhibitory** **Dilution**
Alcoholic extract of Thyme	1:16
Alcoholic extract of Black pepper	1:4
Alcoholic extract of Red pepper	1:4
Thyme oil	1:32
Black pepper oil	1:16
Red pepper oil	1:16

**Table 4:** Determination of minimum inhibitory dilution of extracts for *E. coli O157:H7*

**Plant extracts**	**Minimum Inhibitory** **Dilution**
Alcoholic extract of Thyme	1:64
Alcoholic extract of Black pepper	1:4
Alcoholic extract of Red pepper	1:4
Thyme oil	1:64
Black pepper oil	1:32
Red pepper oil	1:32

MID was determined as the lowest dilution of the tested plants which inhibited the growth of *Escherichia coli O157: H7 *and *Staphylococcus aureus *in the ranges of 1:4-1:64*.* The results showed that they play a significant inhibitory role in growing those bacteria. The results also confirm that, those extracts and oils can prevent the production of DNase enzyme at concentrations lower than the minimum inhibitory dilution in the ranges of 1:16-1:64. (Table 5, 6). 

**Table 5:** Effect of sub-minimal inhibitory dilutions of alcoholic extract on DNase activity

**Samples**	**Black pepper**			**Red pepper**			**Thyme**	
	1:16	1:32	1:64	1:16	1:32	1:64	1:32	1:64
**DNase activity**	**-**	**-**	**-**	**-**	**-**	**-**	**-**	**-**

**Table 6:** Effect of sub- minimal inhibitory dilutions of essential oils on DNase activity

**Samples**	**Black pepper**		**Red pepper**		**Thyme**
	1:32	1:64	1:32	1:64	1:64
**DNase activity**	**-**	**-**	**-**	**-**	**-**

In the last few decades, there has been exponential growth in the field of herbal drugs. It is getting popularized in developing and developed countries owing to its natural origin and lesser side effects. Even though pharmacological industries have produced a number of new antibiotics in the last three decades, resistance to these drugs by microorganisms has increased. In general, bacteria have the genetic ability to transmit and acquire resistance to drugs, which are utilized as therapeutic agents. Extracts of plants contain variety of phenolic compounds and essential oils which may inhibit the growth of some microorganisms. In the last few years, antimicrobial properties of plants essential oils (EOs) have been investigated through several observations and clinical studies which purpose them as potential tools to overcome the microbial drug resistance problem (15). Some previous studies demonstrated that *Escherichia* *coli, Pseudomonas aeruginosa*, *Staphylococcus* *aureus *and *Yersinia enterocolitica *are sensitive to Thyme extract (16). It was known that thymol and carvacrol, two major components of Thyme extract, are both effective against *E.coli *and carvacol is more efficient (17). It has been revealed that Thyme essential oil administration can strongly inhibit the activity of *Staphylococcus* *aureus*, *Bacillus subtilis *and *Escherichia coli *(18). Our phytochemical analysis also indicated that administered Thyme extract was rich in thymol and carvacol. On the other hand, our results stated that Red pepper can inhibit the growth of *Escherichia coli *and *Staphylococcus aureus*. Traditionally, Red pepper boiled extract was administered during enteritis and diarrhea. Some recent studies also clarified the efficacy of Red pepper on diarrhea caused by *E.coli *(19). According to our phytochemical analysis the main component of this extract was galic acid. It was indicated that galic acid can inhibit the growth of some gram positive bacteria. It was also shown that, 18 strains of *Staphylococcus* *aureus *did not coagulate plasma containing tanic acid, galic acid and ellagic acid after incubation for 24 h at 37 °C (20). It seems that a part of these effects of Red pepper may be due to its galic acid component.

 In present study alcoholic extract and essential oil of Black pepper were effective against growth/activity of *Escherichia coli *and *Staphylococcus aureus *and piperine and oleic acid were major components in phytochemical analysis. Previous studies also reported that different extracts of Black pepper displayed excellent inhibition on the growth of gram positive and gram negative bacteria. The major components of Black pepper, piperine (74.34%) and oleic acid (40.67%), were considered as powerful antibacterial substances. Some studies have indicated their inhibitory effects on gram positive and negative bacteria (10).

 Furthermore, in our study extracts and essential oils of Black and Red pepper and Thyme could inhibit the release of *Staphylococcus* *aureus *DNase enzyme in the dilutions lower than minimum inhibitory dilution (MID). Subminimal inhibitory dilutions of Thyme essential oils and alcoholic extracts on DNase activity were more effective. The applied studies on the antimicrobial activity of Black and Red pepper and Thyme showed different results. These conflicting results in antimicrobial activity of these plants could be due to differences in their chemical components. It has also reported that the samples collected from different geographic origin with different climates and vegetation show different antimicrobial activities (10, 11). Therefore, it seems that such plants need to be investigated further about their properties, safety and efficiency for clinical application. Moreever, because of rapidly growing drug resistance of pathogenic bacteria, these extracts and essential oils are suggested for further research the for treatment of infectious diseases caused by resistant microbes.
